# Failed induction of labor and its associated factors in Ethiopia: A systematic review and meta-analysis

**DOI:** 10.1016/j.heliyon.2021.e06415

**Published:** 2021-03-08

**Authors:** Abenezer Melkie, Dagne Addisu, Maru Mekie, Enyew Dagnew

**Affiliations:** Debre Tabor University, College of Health Sciences, Department of Midwifery, Ethiopia

**Keywords:** Failed induction, Induction, Bishop score, Factors, Prevalence, Ethiopia

## Abstract

**Introduction:**

Failed induction increased maternal morbidity and mortality due to the associated complication which comes with cesarean section such as post partum hemorrhage and sepsis. The reports of previous articles on the proportion and associated factor of failed induction were variable and inconsistent. Therefore, this meta-analysis found out that the pooled proportion of failed induction and its associated factors in Ethiopia.

**Methods:**

Systematic search was done by online databases (Pub Med, Web of Science, Google scholar and HINARI, and Ethiopian universities digital libraries). Unpublished studies that are found in the Ethiopian universities’ digital libraries were used for this systematic review and meta-analysis study. Data were entered into Microsoft Excel and then exported to STATA 11 version statistical software for analysis. Heterogeneity assessed using the I^2^ statistic. The pooled proportion of failed induction and the odds ratio (OR) with a 95% confidence interval was showed using forest plots.

**Result:**

The overall proportion of failed induction was 23.58 % (95% CI: 13.72–33.44). Unfavorable Bishop Score [OR = 4.45, 95CI:2.44,8.12 ] intermediate Bishop Score [OR = 8.87, 95CI:4.62,17.05 ] and being primiparous woman [OR = 3.04, 95CI:1.74,5.53 ] were factors associated with failed induction of labour.

**Conclusion:**

The prevalence of failed induction was high in Ethiopia. Unfavorable Bishop Score, intermediate Bishop Score, and primiparous were significantly associated with failed induction. Proper pelvis assessment for Bishop Score will be considered prior to initiating the induction of labor. Beside to this, the health professionals shall be aware of the relevance of cervical ripening for intermediate and unfavorable Bishop Score for pregnant women's before induction of labor.

## 1 Introduction

Induction is commencement of uterine contractions by using uterotonic drugs prior to the onset of spontaneous labor with or without ruptured membranes [1]. The artificial stimulation of uterus before the beginning natural labor helps to improve feto-maternal outcome [2]. Induction of labor shall be done for medical, obstetrical, or other indications & the expected benefits should outweigh its disadvantage [2].

Induction has an impact on the birth experience of women than spontaneous labor and always not end with vaginal delivery [3].Induction of labor has two outcomes; that is either failure or success [3]. The diagnosis of failed induction is still controversial in the clinical setting and there are different diagnostic criteria for different setting [4]. Failed induction is unable to get adequate uterine contraction and poor cervical changes after 6–8 h of oxytocin administration with the use of maximum dose and drops for at least one hour [5].

Feto-maternal morbidity and mortality have been one of the most health problems and challenges that concern the globe over the years, especially in sub-Saharan Africa [6]. In each day greater than 800 maternal deaths happen globally and 99% of these deaths are occurring in developing countries [7]. One-third of these deaths occur in South Asia and more than half occur in sub-Saharan Africa, significantly contributed by six countries including Ethiopia [7]. Findings revealed that the failed induction of labor increased the rate of operative delivery, Postpartum hemorrhage, and prolonged maternal and neonatal hospital stay [8].

The rate of induction is 20% at the global level; up to 25% in developed countries [7]. The recent world health organization (WHO) worldwide Survey in Africa revealed that the rate of induction varies from 1.4% to 6.8% which low in comparison to WHO recommendation [6].

According to the studies which were done in Saudi Arabia, Nepal, Zambia, and Zimbabwe the prevalence of failed induction showed that 16.3%, 18%, 13.4%, and 24.9% respectively [2, 9, 10, 11]. The prevalence of failed induction varies across different settings in Ethiopia which is about 19.7% in Dessie referral hospital, 21.4% in Jimma specialized hospital, 37.4% in Wolidia hospital, 17.3% in Hawassa public hospitals, 40.3% in Addis Abeba Army hospital [3, 5, 12, 13, 14].

Known predictors of failed induction of labor are being primiparous mother, unfavorable bishop score, fetal macrosomia, a high body mass index, and advanced maternal age [9, 15]. Assessing the proportion and associated factors of failed induction in Ethiopia is mandatory because maternal deaths did not reduce considerably to meet the Sustainable Development Goal for maternal health, and was estimated at 412 maternal deaths/100,000 live births in 2016 [16].

Even though there are various studies done to assess the proportion and associated factors of failed induction, there is a lack of data to show the proportion and associated factors of failed induction at the national level. Besides, the proportion and factors associated with failed induction were inconsistent in primary studies. Therefore, this systematic review and meta-analysis intended to approximate the overall proportion of failed induction and its associated factors in Ethiopia.

## 2 Methods

### 2.1 Searching strategies

International databases (i.e. Pub Med, Web of Science, Google scholar and HINARI) were used to search published studies. Ethiopian university digital libraries also used to search unpublished studies. Studies published between September 20, 2005 and July 29, 2020 G.C were included in this systematic review and Meta -analysis. Searching terms used for Google scholar were "Prevalence", "magnitude", "proportion", "failed induction", "associated factors", "determinants","Ethiopia". Searching terms were established using the Boolean operator “and” and “or”. Search details for Pub Med were ("epidemiology" [Subheading] OR "epidemiology" [All Fields] OR "prevalence" [All Fields] OR "prevalence" [MeSH Terms] OR "magnitude" [All Fields]) AND failed [All Fields] AND induction [All Fields] AND associated [All Fields] AND factors [All Fields] AND ("Ethiopia" [MeSH Terms] OR "Ethiopia" [All Fields])

### 2.2 Inclusion criteria

This systematic review and meta-analysis included articles that meet the following criteria: study that reports prevalence or proportion and/or associated factors of failed induction, Both gray kinds of literature and published articles that were written with the English language,Observational study designs (case-control and cross-sectional study designs) that report prevalence or proportion and/or associated factors of failed induction and studies that were published from September 20, 2005, to July 30, 2020.

### 2.3 Exclusion criteria

Literature that was not fully accessible and failure to reply from the corresponding authors within three weeks of contact by email were excluded. Similarly, articles not reporting the outcome of interest were excluded.

### 2.4 Study selection and quality assessment

First, all relevant searched articles were exported to Endnote version 6 reference manager. Then after the careful evaluation was done and duplicated studies were removed. Titles and abstracts were screened by four reviewers (AM, DA, MM and ED) independently. The quality of the studies was assessed using the Newcastle - Ottawa quality assessment Scale (NOS) for cross-sectional and case-control studies [17]. The difference between the reviewer's views for inclusion and exclusions of articles was solved by discussion. We used the following criteria to appraise cross-sectional studies: (1) Representativeness of the sample; (2) Non-respondents; (3) Ascertainment of the exposure (risk factor); (4)The subjects in different outcome groups are comparable, based on the study design or analysis; Confounding factors are controlled; (5) Assessment of the outcome; (6) Statistical test. For case-control studies we have used the following criteria: (1) adequate case definition; (2) Representativeness of the cases; (3) Selection of Controls; (4) Definition of Controls; (5) Comparability of cases and controls based on the design or analysis; (6) Ascertainment of exposure; (7) Same method of ascertainment for cases and controls; (8) Non-Response rate. Studies were considered as high-quality when articles scored ≥7 points out of 9 for cross-sectional and ≥9 points out of 10 for case-control studies.

### 2.5 Measurement of outcome variables

Failed induction of labor is the first outcome for this systematic review and meta-analysis study. Identifying associated factors of failed induction was the second outcome for this study. Intermediate Bishop Score, unfavorable Bishop Score, primiparous and age greater than 30 years were predictor factors included in this review. The Adjusted odds ratio was taken from the reports of primary studies.

#### 2.5.1 Failed induction of labor

It is unable to get adequate uterine contraction and having of poor cervical changes after 6–8 h of oxytocin induction with the use of highest dose and drops for at least one hour [18].

#### 2.5.2 Favorable bishop score

A cervical assessment score ≥9 were considered as having favorable Bishop Score [18].

#### 2.5.3 Intermediate Bishop Score

A cervical assessment score 5–8 were considered as having intermediate Bishop Score [18].

#### 2.5.4 Unfavorable Bishop Score

A cervical assessment ≤4 were considered as unfavorable Bishop Score [18].

### 2.6 Data extraction

Abenezer Melkie (AM) and Dagne Addisu (DA) autonomously extracted all necessary data using a standardized data extraction format. The data extraction format included primary author, publication year, and country of the study, study design, sample size, factor and prevalence.

### 2.7 Data analysis

Data were entered into Microsoft Excel and then export to STATA Version 11 statistical software for analysis. Heterogeneity between reported prevalence was assessed by calculating the p-values of I^2^ statistics [19].Heterogeneity was observed between the studies while assessing the polled prevalence of failed induction in Ethiopia (I^2^ = 97.9%, P ≤ 0.01). Therefore, random effect models were used to assess the pooled proportion of failed induction in Ethiopia [20]. Subgroup analysis was done to recognize possible source heterogeneity by using sample size categories and the region where the study was conducted. Funnel plot and eggers regression test used to assess publication partiality between the studies [21, 22].

## 3 Result

### 3.1 Search result

**Figure 1:**
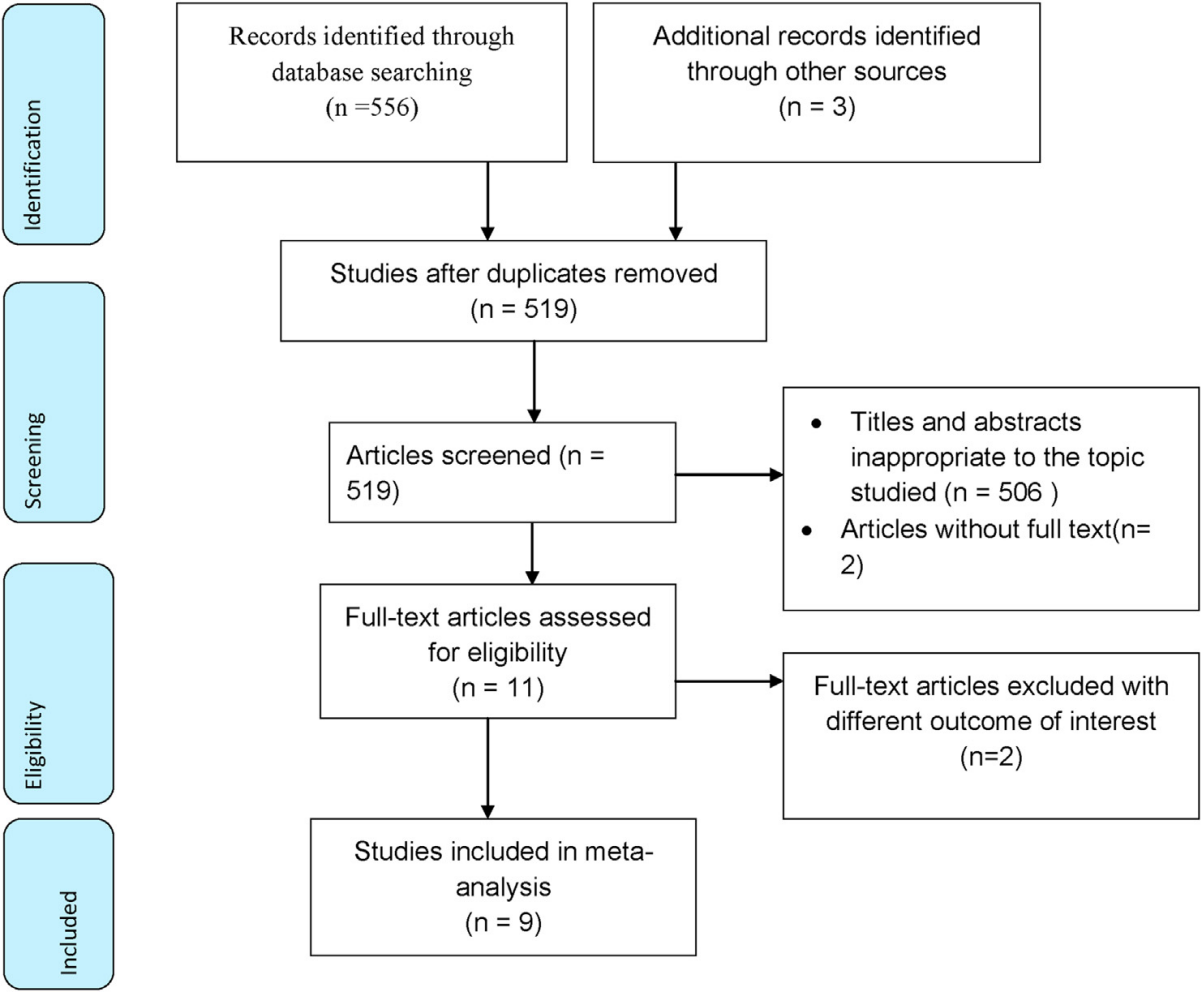
Flow chart of study selection for systematic review and meta-analysis of failed induction and associated factors in Ethiopia.

A sum of 559 studies regarding the proportion and associated factors of failed induction Ethiopia were searched from these databases: Pub Med, Web Science, HINARI, Google Scholar, and Ethiopia university digital libraries. All searched articles were exported to the endnote and then 40 were removed due to duplication. Five hundred nineteen studies were screened for eligibility, relevance, accessibility, and outcome of interest. Accordingly, 506 studies excluded due to inappropriate titles, and 2 studies due to inaccessible full text. Two articles were removed due to different outcomes of interest. Finally, 9 studies included in this systematic review and meta-analysis (Figure 1).

### 3.2 Characteristics of included studies

In this systematic review and meta-analysis; a total of nine studies with 2861 sample sizes were included. Based on study design; eight studies were cross-sectional [3, 5, 12, 14, 23, 24, 25] and one was a case-control study [26]. The studies were published between 2015 and 2020 G.C. In this study four regions and one administrative the city were represented. Three studies were from the Amhara region [3, 14, 26], two studies from SNNPR(Southern nation nationalities people region) [23, 24], one study from the Oromia region [5], one study from the Tigray region [25], and two studies from Addis Ababa city [12, 27] (Table 1).

**Table 1:** Descriptive summery of nine studies incorporated in the meta-analysis of prevalence and associated factors of failed induction in Ethiopia.

Author	Publication year	Region	study area	study design	sample size	Prevalence (%)	Quality Of the study
Dilnessa et al	2019	Amhara	Dessie referal hospital	cross-sectional	319	19.74	Low risk
Girma et al	2018	Ormiya	Jima referal hospital	cross-sectional	280	21.42	Low risk
Wodaje et al	2018	Woliya	Woldia hospital	cross-sectional	380	37.36	Low risk
Huresia et al	2015	SNNPR	Hawassa hospital	cross-sectional	294	17.34	Low risk
Melkie et al	2018	Amhara	At referal hospitals in amhara region	case-control	336	-	Low risk
Hilufsara	2015	AddisAbeba	Armey hospital	cross-sectional	347	40.34	Low risk
Lueth et al	2020	Tigray	Mekelle hospital	cross-sectional	346	7.22	Low risk
Bekru et al	2018	SNNPR	Otona hospital	cross-sectional	347	40.34	Low risk
Aluk et al	2018	Addis Abeba	St.pauls millenum hospital	cross-sectional	212	5.66	Low risk

### 3.3 Publication bias

**Figure 2:**
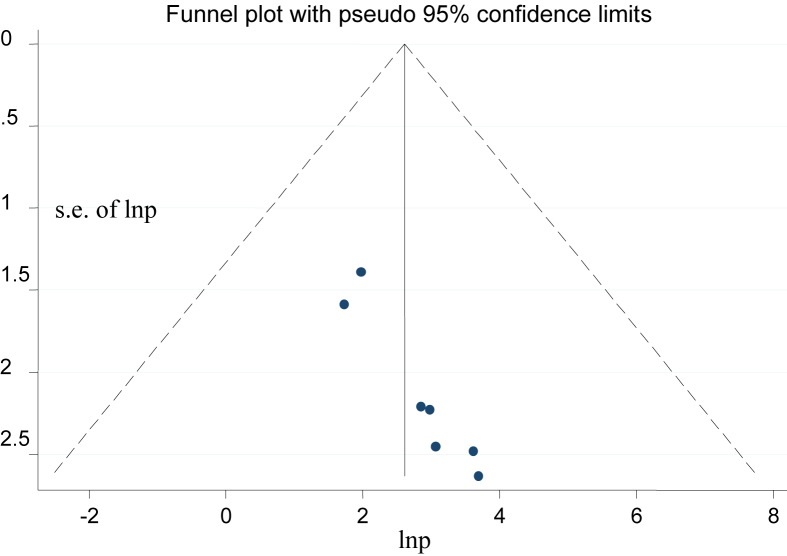
Funnel plots of the prevalence of failed induction in Ethiopia.

**Figure 3:**
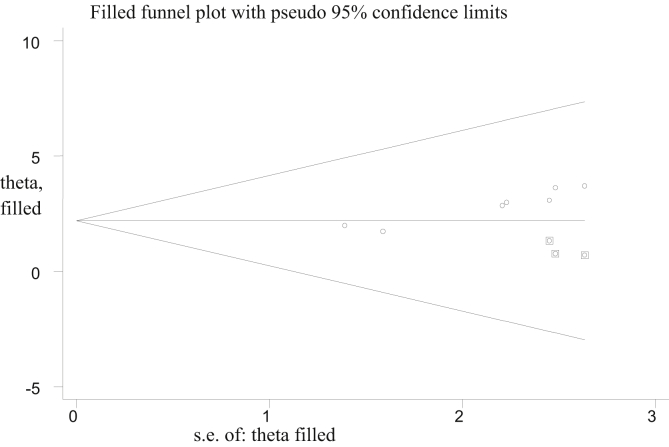
Funnel plot of prevalence of failed induction and associated factors after publication bias was adjusted by trim fill analysis.

The funnel plot and egger's regression test were used to review the possibility of publication bias across the studies [21, 22]. The test result showed that publication bias was observed between the studies (egger's regression test p-value = 0.001). Also, asymmetry was seen in the funnel plot (Figure 2). Therefore, Duval and Tweedie's Trim and Fill analyses were done to overcome publication bias across the studies. The corrected pooled prevalence of failed induction after adding four studies with fill and trim analysis was 10.99%. Accordingly, publication bias was corrected when four studies were included in the funnel plot by trim fill analysis (Figure 3).

### 3.4 Prevalence of failed induction in Ethiopia

**Figure 4:**
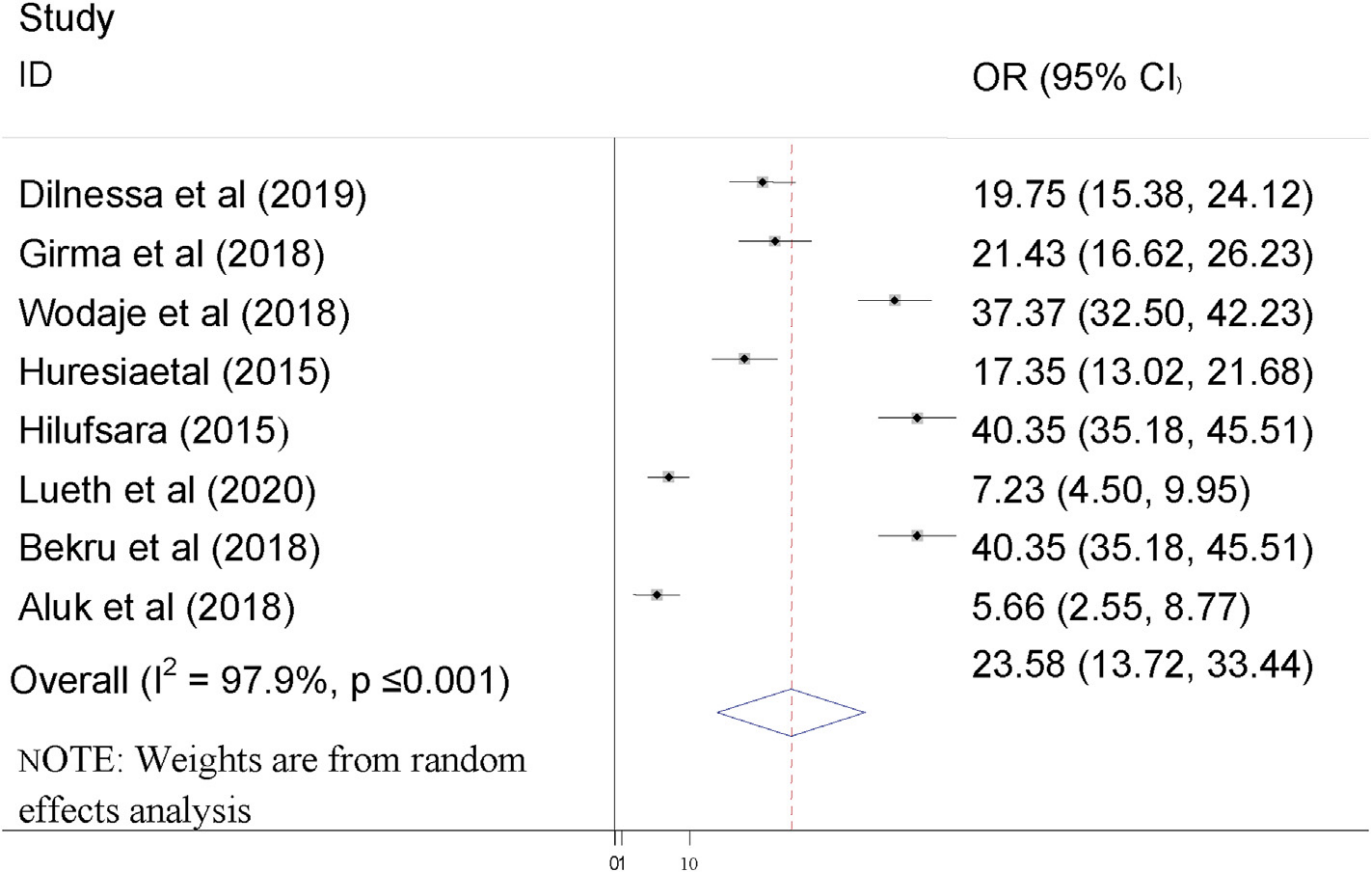
Forest plot showing pooled prevalence of failed induction in Ethiopia.

The raw pooled prevalence of failed induction was 23.58% with 95%CI (13.72, 33.44). I-squared statistics revealed considerable heterogeneity across the included studies (I^2^ = 97.9%, P ≤ 0.01). As a result, random effect models were used to determine the pooled prevalence of failed induction in Ethiopia. From the included studies, the highest prevalence of failed induction was reported by Hilufsara and Bekru et al which was 40.35% [12, 24] and the lowest was 7.2% which was reported by Lueth et al [25] (Figure 4).

### 3.5 Subgroup analysis

To identify the possible source of heterogeneity subgroup analysis was done based on sample size category and by the region where the studies were conducted. However, none of these variables were significant. According to the sample size category, the highest proportion was Observed in studies that had a sample size of >320 with 31.25% with CI of (11.65, 50.85). Based on the region, the highest proportion of failed induction was seen in SNNRP with a prevalence of 28.8% (6.26, 51.34) followed by the Amhara and Tigray region with the prevalence of 28.53 % (11.26, 45.79) and 23.58% (13.72, 33.44) respectively (Table 2). Studies incorporated in this systematic review and meta-analysis were done in regional hospitals of Ethiopia (Table 3).

**Table 2:** Subgroup analysis of proportion of failed induction in Ethiopia.

Variables	Characteristics	Included studies	Sample size	Prevalence with (95%)
By region	Amhara	2	699	28.53% (11.26,45.79)
	Oromia	1	280	21.43% (16.62, 26.23)
	Tigray	1	346	23.58% (13.72,33.44)
	Addis Abeba	2	559	22.94 % (11.05, 56.93%)
	SNNPR	2	641	28.80% (6.26,51.34)
By sample size	>320	4	1420	23.58% (13.72,33.44)
	≤320	4	1105	15.93% (7.96, 23.90)

**Table 3:** Studies setting, induction protocol, and the definition of failed induction for prevalence and associated factors of failed induction in Ethiopia.

Author	Study setting	Induction protocol		Definition of failed induction
		Primigravida	Multigravida	
Dilnessa et al	Dessie referal hospital	5IU	2.5IU	It is unable to get adequate uterine contraction and having of poor cervical changes after 6–8 h of oxytocin induction with the use of highest dose and drops for at least one hour
Girma et al	Jima referal hospital	5IU	2.5IU	It is unable to get adequate uterine contraction and having of poor cervical changes after 6–8 h of oxytocin induction with the use of highest dose and drops for at least one hour
Wodaje et al	Woldia hospital	5IU	2.5IU	It is unable to get adequate uterine contraction and having of poor cervical changes after 6–8 h of oxytocin induction with the use of highest dose and drops for at least one hour
Huresia et al	Hawassa hospital	5IU	2.5IU	It is unable to get adequate uterine contraction and having of poor cervical changes after 6–8 h of oxytocin induction with the use of highest dose and drops for at least one hour
Melkie et al	Amhara region referal hospitals	5IU	2.5IU	It is unable to get adequate uterine contraction and having of poor cervical changes after 6–8 h of oxytocin induction with the use of highest dose and drops for at least one hour
Hilufsara	Armey hospital	5IU	2.5IU	It is unable to get adequate uterine contraction and having of poor cervical changes after 6–8 h of oxytocin induction with the use of highest dose and drops for at least one hour
Lueth et al	Mekelle hospital	5IU	2.5IU	It is unable to get adequate uterine contraction and having of poor cervical changes after 6–8 h of oxytocin induction with the use of highest dose and drops for at least one hour
Bekru et al	Otona hospital	5IU	2.5IU	It is unable to get adequate uterine contraction and having of poor cervical changes after 6–8 h of oxytocin induction with the use of highest dose and drops for at least one hour
Aluk et al	St.pauls millenum hospital	5IU	2.5IU	It is unable to get adequate uterine contraction and having of poor cervical changes after 6–8 h of oxytocin induction with the use of highest dose and drops for at least one hour

### 3.6 Factors associated with failed induction

In this systematic review and meta-analysis; factors associated with failed induction were unfavorable bishop score 4.45 (95CI:2.44, 8.12), intermediate bishop score 8.87 (95CI:4.62, 17.05) and primiparous 3.04 (95CI:1.74, 5.33).

### 3.7 The association between failed induction and unfavorable Bishop Score

**Figure 5:**
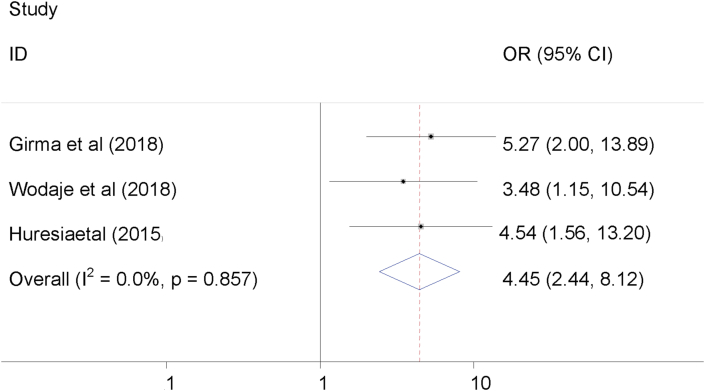
Pooled odds ratio of the association between unfavorable Bishop Score and failed induction.

The association between unfavorable Bishop Score and failed induction was examined using three studies [5, 14, 23]. The finding indicated that a significant association was observed between failed induction and unfavorable Bishop Score. Women's had unfavorable Bishop Score were 4.54 times more likely to develop failed induction as compared to their counterparts. There was no heterogeneity across the studies (I^2^ = 0). Hence, fixed-effect the model was used to determine the relationship (Figure 5).

### 3.8 The association between failed induction and intermediate Bishop Score

**Figure 6:**
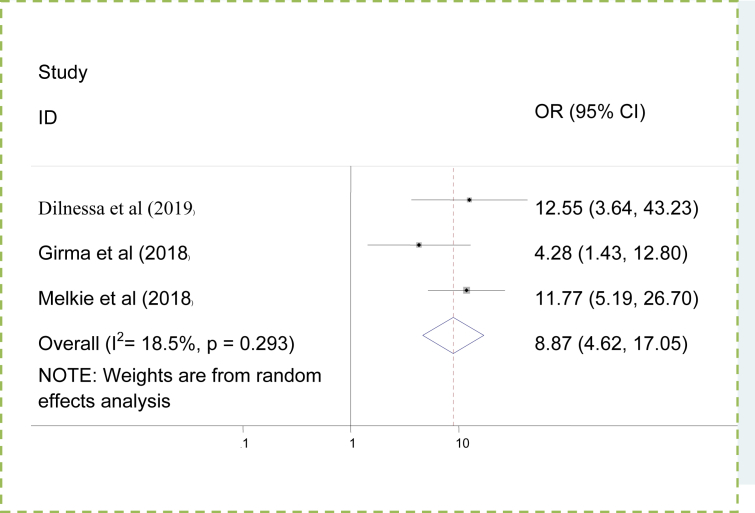
Polled odd ratio of the association between intermediate Bishop Score and failed induction.

The association between intermediate Bishop Score and failed induction was examined using three studies [3, 5, 26]. The finding showed that women who had intermediate Bishop Score were 8.87 times more likely to develop failed induction as compared to those who had favorable Bishop Score [OR = 8.87,95%CI, 4.62,17.05]. There was minimal heterogeneity across the studies (I^2^ = 18.5%). Hence; the random effect model was used to determine the association (Figure 6).

### 3.9 The association between failed induction and primiparous

**Figure 7:**
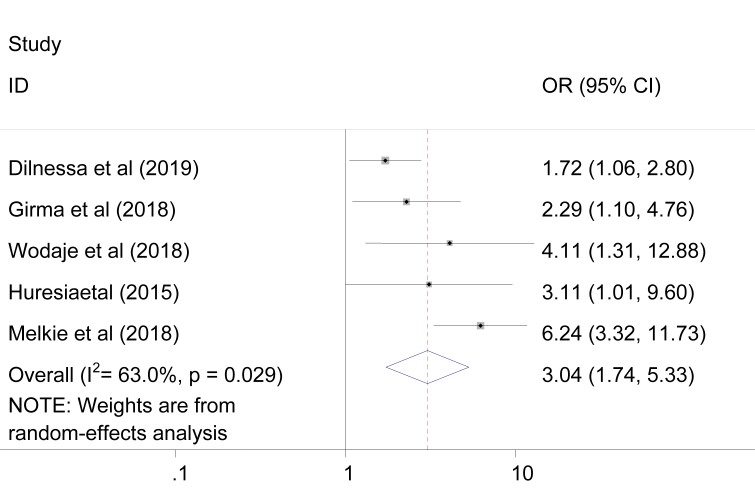
Pooled odd ratio of the association between primiparous and failed induction.

Five studies were used to assess the association between primiparous and failed induction [3, 5, 14, 23, 26]. The finding revealed that the likely hood of developing failed induction was 3.04 times more likely in primiparous women as compared to multiparous women [OR = 3.04,95%CI = 1.74,5.33]. In this review moderate heterogeneity was observed between the studies (I^2^ = 63%). Therefore, a random effect model was applied to measure the association between primiparous and failed induction (Figure 7).

### 3.10 The association between failed induction and maternal age

**Figure 8:**
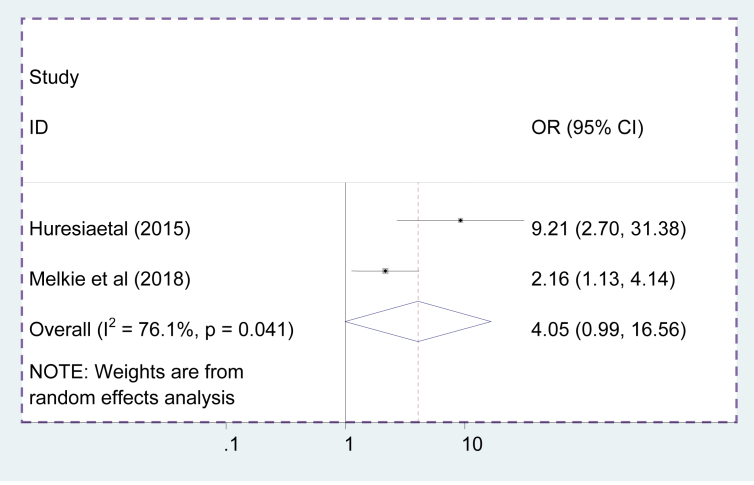
Pooled odds ration of the association between maternal age and failed induction.

Two primary studies reported that maternal age >30 years were associated with failed induction. However, there was no association between maternal age and failed induction in this systematic reviewand meta-analysis study [OR = 4.05, 95%CI = 0.99, 16.56] (Figure 8).

## 4 Discussion

This review was conducted to estimate the overall proportion of failed induction and associated factors in Ethiopia. The overall proportion of failed induction among women who had an induction in Ethiopia was 23.58 % (95% CI: 13.72, 33.44). The prevalence of failed induction seen in the present study was in line with the studies done in Australia (15.2%), Pakistan (22%), in a health resource-poor setting (24.1%), and Saudi Arabia (16%) [9, 15, 28, 29].

The pooled proportion of the current study was lower than a study done in India with a proportion of 50.5% [30]. The discrepancy might be due to the variation in study area, induction protocol, and the definition of failed induction.

The sub-group analysis showed that the prevalence of failed induction among women who had induction slightly varies across regions. The uppermost prevalance of failed induction was seen in SNNPR (28.80%) and the Amhara region (28.53%). The lowest prevalence of failed induction was observed in the Oromia region (21.43%). This could be institutional delivery coverage in Oromia region is 19% [16]which is low in comparison to the Amhara region 27% [16] and SNNPR 26% [16]which affects the prevalence of women's who had failed induction. The prevalence of failed induction across different hospitals of Ethiopia were; 19.7% in Dessie referral hospital, 21.4% in Jimma specialized hospital, 37.4% in Wolidia hospital, 17.3% in Hawassa public hospitals, and 40.3% in Addis Abeba Army hospital [3, 5, 12, 13, 14]. This high prevalence of failed induction associated with operative delivery and its associated complication [2] and might raise the maternal deaths in Ethiopia.

The present study showed that having unfavorable Bishop Score were 4.54 times more likely to got failed induction as compared to those who had a favorable Bishop Score. This result was aggregated by previous studies done in Great Britain, Spain, and Nigeria [4, 31, 32]. This could be explained by the reliance of induction on cervical condition. If pregnant women's cervix is not well repined their might be high failures of induction due to poor progress of the cervix. The current finding showed that significant association was observed between failed induction and intermediate Bishop Score. Having intermediate Bishop Score were 8.87 times more likely to develop failed induction as compared their counter parts. The possible explanation could be poor ripening of the cervixes before initiations of induction and increasing high-risk obstetric cases that might lead to unplanned induction.

This review revealed that a considerable association was seen between failed inductions and primiparous mothers. The likelihood of developing failed induction was 3.04 times prone in primiparous women as compared to multiparous mothers. This discovery agrees with the studies reported in India, Pakistan, and Saudi Arabia [9, 11, 15]. This could be primiparous women might have less response to ripening methods, and having untested pelvis.

### 4.1 Limitation

This meta-analysis and systematic review might not exactly the national figure of failed induction because of inadequate number of studies and under-representation of the different regions in the country.

## 5 Conclusion

The proportion of failed induction was high in Ethiopia. Unfavorable Bishop Score, intermediate Bishop Score, and primiparous were significantly associated with failed induction. Proper pelvis assessment for Bishop Score will be considered prior to initiating the induction of labor. Beside to this, the health professionals shall be aware of the relevance of cervical ripening for intermediate and unfavorable Bishop Score for pregnant women's before induction of labor.

## Declarations

### Author contribution statement

All authors listed have significantly contributed to the development and the writing of this article.

### Funding statement

This research did not receive any specific grant from funding agencies in the public, commercial, or not-for-profit sectors.

### Data availability statement

Data will be made available on request.

### Declaration of interests statement

The authors declare no conflict of interest.

### Additional information

No additional information is available for this paper.
